# Intracranial Subdural Fluid Accumulation Associated with a Choroid Plexus Carcinoma in a Dog

**DOI:** 10.3390/vetsci10010024

**Published:** 2022-12-30

**Authors:** Nina Schneider, Andreas Blutke, Kaspar Matiasek, Birgit Parzefall

**Affiliations:** 1 Small Animal Clinic Oberhaching, Bajuwarenring 10, 82041 Oberhaching, Germany; 2 Institute of Veterinary Pathology, Center for Clinical Veterinary Medicine, Ludwig-Maximilians Universität München, Veterinärstr. 13, 80539 Munich, Germany

**Keywords:** choroid plexus tumor, cysts, diffuse, meningeal, metastasis

## Abstract

**Simple Summary:**

Choroid plexus tumors are commonly described as intraventricular mass lesions and account for 7–10% of intracranial, primary tumors in dogs. This paper reports about an unusual case of a choroid plexus carcinoma in a 3-year-old Shetland sheepdog that presented for slowly progressive lethargy, vision impairment and cognitive deficits. On magnetic resonance imaging, a subdural fluid accumulation overlying and compressing the left parietotemporal lobe as well as multifocal changes consisting of cyst-like lesions, supposed intra-axial brain lesions and generalized meningeal changes were identified. Cerebrospinal fluid analysis showed a mononuclear pleocytosis with negative results for infectious agents. The dog was treated with prednisolone followed by burr hole craniotomy with puncture of the subdural fluid accumulation and subsequent chemotherapy that resulted in temporary improvement. The dog deteriorated again and was therefore euthanized. Post-mortem examination revealed a diffuse, highly invasive choroid plexus carcinoma that involved the entire central nervous system. In conclusion, a choroid plexus carcinoma should be considered as a possible cause of a subdural fluid accumulation even in the absence of an intraventricular mass lesion.

**Abstract:**

Choroid plexus tumors are commonly described as intraventricular mass lesions and account for 7–10% of intracranial, primary tumors in dogs. A 3-year-old Shetland sheepdog was presented with a history of slowly progressive lethargy, vision impairment and cognitive deficits. On magnetic resonance imaging, a subdural fluid accumulation (SFA) overlying and compressing the left parietotemporal lobe as well as multifocal changes consisting of cyst-like lesions, supposed intra-axial brain lesions and mild, multifocal meningeal thickening and generalized contrast enhancement were identified. Cerebrospinal fluid (CSF) analysis showed a mononuclear pleocytosis with negative results for infectious agents. The dog was treated with prednisolone followed by burr hole craniotomy with puncture of the SFA, which macroscopically appeared to be CSF-like fluid. After initial improvement, the dog deteriorated despite continuation of prednisolone and cytarabine therapy and was euthanized four weeks after surgery. Histopathology was consistent with a disseminated, neuroinvasive choroid plexus carcinoma (CPC) that involved the entire neuroaxis including the meninges of the brain and spinal cord. Immunohistochemical examination showed a strong Kir7.1 and a heterogenous cytokeratin-immunoreactivity in neoplastic cells. In conclusion, a CPC should be considered as a possible cause of a SFA even in the absence of an intraventricular mass lesion.

## 1. Introduction

Choroid plexus tumors (CPTs) account for 7–10% of intracranial, primary tumors in dogs [1,2] and are usually described as intraventricular mass lesions, which most commonly occur in the fourth ventricle [2,3] and rarely within the cerebellomedullary pontine angle [4]. They are commonly accompanied by hydrocephalus, which can either be rostral or caudal to the mass lesion [3] and which is thought to arise secondary to obstruction of the ventricular system or due to impairment of cerebrospinal fluid (CSF) production and absorption [5,6]. CPTs occur mainly in middle-aged to older dogs with a median age of seven years [3] though a range of 1–14 years has been reported in literature [2,3]. In contrast, CPTs in humans mostly affect infants with 13–26% occurring within the first year of life [7,8]. 

The classification of CPTs in veterinary medicine based on the International World Health Organization histological grading of tumors of the central nervous system (CNS) for humans [3] subdivides CPTs into choroid plexus papilloma (CPP), atypical CPP and choroid plexus carcinoma (CPC) that represent grade I, II and III, respectively [9], and are of high relevance as a prognostic factor in humans [10]. Metastasis within CSF spaces are common in CPCs and have been identified in up to 45–63% of humans with CPC on magnetic resonance imaging (MRI) [8,11] and in 53% of dogs on post-mortem examination [3]. 

CPTs are one of the most important differential diagnoses for an intraventricular neoplasia in dogs and typical neuroimaging features include a strongly contrast-enhancing mass mostly within the fourth ventricle accompanied by hydrocephalus internus and periventricular edema [12]. However, although rare, unusual cases of CPTs presenting without an intraventricular mass on MRI have been reported in dogs [13,14] that can make ante-mortem diagnosis challenging. Here, we describe the clinical, imaging, histopathological, and immunohistochemical characteristics of a diffuse CPC in a dog that was associated with a subdural fluid accumulation, cyst-like lesions and disseminated meningeal involvement in the absence of an intraventricular mass lesion. 

## 2. Case Presentation

A 3.5-year-old, female neutered Shetland sheepdog presented with a 6-month history of slowly progressive lethargy, disorientation and vision impairment that was suspected by the owner. Physical examination was unremarkable except for a body condition score of 8/9. Ophthalmological examination confirmed that the dog was blind in the left eye (OS), but no ophthalmological cause could be identified and therefore a neurological examination was recommended. 

On neurological examination, the dog was mildly obtunded; ran into obstacles when blindfolding the right eye; and had a normal gait, postural reactions and spinal reflexes. Anisocoria with a mydriatic pupil OS was noted as well as an absent menace response, dazzle reflex and pupillary light reflex OS, positional horizontal nystagmus with the fast phase to the right side and discomfort on palpation of the head and cervical spine. Based on these findings, the neuroanatomical localization was multifocal central nervous system (CNS) including forebrain, left-sided vestibular and left-sided retina or optic nerve. The main differential diagnoses were inflammatory and neoplastic disease or less likely a degenerative disease or intracranial malformation.

**Figure 1:**
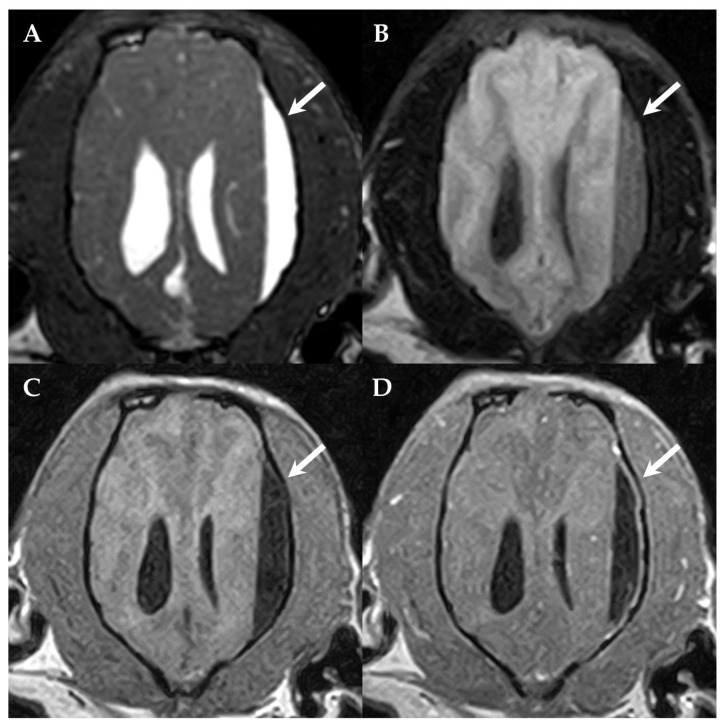
First MRI of the dog’s brain. Dorsal BASG (**A**), FLAIR (**B**) 3DT1W (**C**) and 3DT1W after contrast administration (**D**) showing the subdural fluid accumulation (SFA, arrows) compressing the left parietotemporal lobe and the left lateral ventricle. The SFA is BASG hyperintense (**A**), incompletely compressing on FLAIR (**B**), 3DT1W hypointense and demonstrates a mild contrast-enhancement of the adjacent dura mater (**D**). The left side of the patient is on the right side of the image.

**Figure 2:**
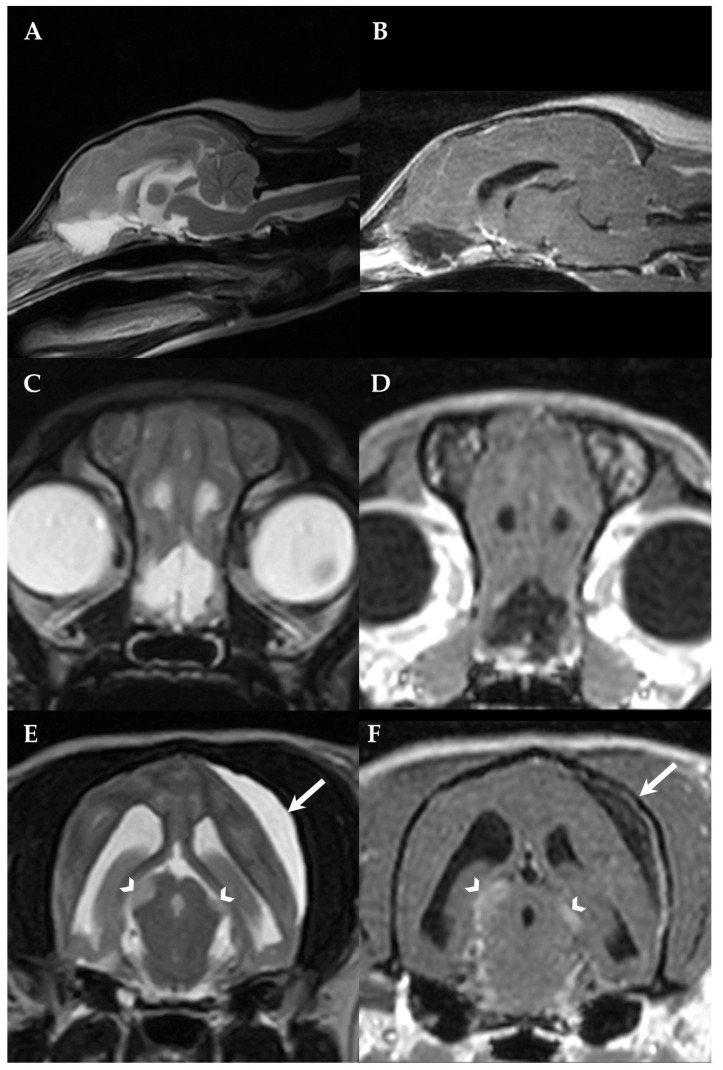
First MRI of the dog´s brain. Sagittal T2W(**A**), sagittal 3DT1W after contrast administration (**B**), transversal T2W (**C**) and transversal 3DT1W after contrast administration (**D**) at the level of the olfactory bulb, transversal T2W (**E**) and transversal 3DT1W after contrast administration (**F**) at the level of the midbrain. Bilateral cyst-like lesions within the olfactory bulb and frontal lobe (**A**–**D**) as well as T2W hyperintense (**E**) and contrast-enhancing (**F**) asymmetrical midbrain lesions were identified (arrowheads). The SFA (arrows) can also be recognized, compressing the left parietotemporal lobe (**E**,**F**). Note the mild generalized meningeal enhancement and thickening (**B**,**D**,**F**). The left side of the patient is on the right side of the image.

Complete blood cell count, serum biochemistry, electrolytes and thoracic radiographs were unremarkable. The dog was premedicated with diazepam 0.1 mg/kg IV (Solupam^®^, Dechra, Raamsdonksveer, The Netherlands) and butorphanol 0.2 mg/kg IV (Morphasol^®^, Dechra, Senden-Bösensell, Germany), the anesthesia was induced with an IV bolus of propofol (Narcofol^®^, CP-Pharma, Burgdorf, Germany) and maintained with isoflurane and oxygen mixture for magnetic resonance imaging (MRI). MRI was performed using the Aperto Lucent 0.4 T scanner (Hitachi Medical Corporation, Tokyo, Japan) and sagittal and transvere T2-weighted (T2W), dorsal fluid-attenuated inversion recovery (FLAIR), dorsal 3-dimensional T1-weighted (3DT1W) before and after IV contrast administration (0.1 mmol/kg bodyweight, gadoterate meglumine (Dotarem^®^; Guerbet, Roissy CdG cedex, Villepinte, France)) including transverse and sagittal reconstructions, dorsal balanced steady-state gradient echo sequence (BASG) and transverse T2* images were obtained. MRI revealed a T2W hyperintense, crescent-shaped lesion within the subdural space overlying the left parietotemporal lobe and causing compression of the adjacent cerebral hemisphere with attenuation of the left lateral ventricle. The lesion was 3DT1W hypointense, incompletely suppressed on FLAIR; had no signal void on T2*; and showed a mild contrast enhancement of the adjacent dura mater, while there was no leptomeningeal pattern or contrast enhancement of the content of the subdural lesion (Figure 1 and Figure 2E,F). 

Additional intra-axial, thin-walled, T2W hyperintense, 3DT1W and FLAIR hypointense lesions were noted within the olfactory bulb and the frontal lobe which were surrounded by T2W and FLAIR hyperintensities consistent with perilesional oedema (Figure 2A–D). Multiple ill-defined supposed intra-axial T2W hyperintense, 3DT1W iso- to hypointense and moderately contrast-enhancing lesions were recognized bilateral asymmetrically affecting the tectum mesencephali with a diameter of 6 × 3 mm on the right and 4 × 2 mm on the left side (Figure 2E,F) and mildly contrast-enhancing 4 × 3 mm lesions within the left lateral geniculate nucleus. A mild generalized meningeal enhancement and a mild, multifocal meningeal thickening were also recognized (Figure 2B,D,F).

CSF analysis showed a mononuclear pleocytosis with a total cell count of 110 cells/μL (reference range 0–5) and an elevated protein of 0.83 g/L (reference < 0.3). The CSF infectious disease panel including polymerase chain reactions (PCRs) for canine distemper virus, *Toxoplasma gondii, Neospora caninum, Bartonella species* and enzyme-linked immunosorbent assay (ELISA) for tick-borne encephalitis virus were negative. Based on these further diagnostic findings, meningoencephalitis or multifocal intracranial neoplasia were suspected to be the most likely differential diagnoses. 

Surgical evacuation of the compressive SFA was recommended but declined by the owner. The dog was treated with dexamethasone 0.4 mg/kg IV once daily (Hexadreson^®^, MSD Animal Health, Unterschleißheim, Germany), clindamycin 20 mg/kg IV twice daily (Clindamycin^®^, Ratiopharm, Blaubeuren, Germany) while awaiting CSF results and until a *toxoplasma gondii* or *neospora caninum* infection could be excluded, pantoprazole 1 mg/kg IV twice daily (Pantoprazol^®^, Hexal, Ljubljana, Slovenia), buprenorphine 0.005 mg/kg IV three times daily (Bupresol Multidose^®^, CP-Pharma, Burgdorf, Germany) and with a continuous rate infusion of lactated Ringer’s solution (Ringer-Infusionslösung^®^, B Braun, Melsungen, Germany) during the time of hospitalization. After four days the dog was discharged and PO treatment was continued consisting of prednisolone 2 mg/kg once daily (Prednisolon^®^, CP-Pharma, Burgdorf, Germany), clindamycin 20 mg/kg twice daily (Cleorobe^®^, Zoetis, Berlin, Germany), omeprazole 1 mg/kg once daily (Omep^®^, Heumann, Madrid, Spain) for its potential to decrease CSF production [15], gabapentin 5 mg/kg three times daily (Gapaliquid^®^, GeriSan, Heppenheim, Germany) and metamizole 40 mg/kg three times daily (Novaminsulfon^®^, Ratiopharm, Blaubeuren, Germany). 

**Figure 3:**
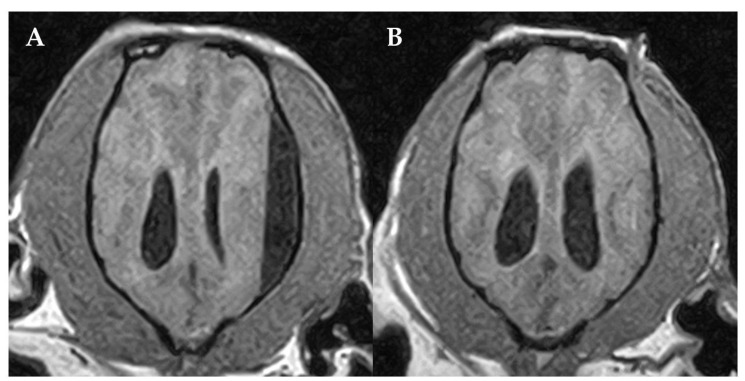
Pre- (**A**) and post-surgical (**B**) MRI of the dog´s brain. Dorsal 3DT1W (**A**,**B**). Post-surgical MRI (**B**) confirmed the resolution of the SFA. The left side of the patient is on the right side of the image.

The dog was presented for re-examination one week after diagnosis with anorexia and neurological deterioration consisting of obtundation to stupor, bilateral blindness, right-lateralized ambulatory tetraparesis and head and cervical pain. Progression of mass effect caused by the SFA was suspected and due to the worsening of the dog, the owner agreed on surgery. The dog was premedicated with diazepam 0.1 mg/kg IV (Solupam^®^, Dechra, Raamsdonksveer, The Netherlands) and methadone 0.3 mg/kg IV (Comfortan^®^, Dechra, AE Bladel, The Netherlands), anesthesia was induced with an IV bolus of propofol (Narcofol^®^, CP-Pharma, Burgdorf, Germany) and maintained with isoflurane and oxygen mixture. Cefazoline (Cefazolin-saar^®^, MiP-Pharma, Blieskastel, Germany) was administered perioperatively at 20 mg/kg IV once. The dog was positioned in sternal recumbency and burr hole craniotomy was performed via a left-sided rostrotentorial approach with puncture of the SFA using a 20-gauge IV catheter, which macroscopically was consistent with CSF-like fluid. Postoperative MRI, obtaining transverse T2W and dorsal 3DT1W images including transverse and sagittal reconstructions, confirmed a resolution of the SFA (Figure 3) except for a thin T2W hyperintense, and 3DT1W hypointense rim. The lateral ventricles were symmetrical, not attenuated anymore and mildly dilated. Additional findings compared to the first MRI were identified including bilateral asymmetrical and diffuse T2W hyperintense, 3DT1W isointense lesions with the grey matter of the parietal lobes and T2W hyperintense, 3DT1W isointense material within the right middle ear and outer ear canal consistent with otitis externa and media. Right-sided bacterial otitis externa was confirmed by otic cytological evaluation. Esophagostomy tube placement because of anorexia was performed under the same anesthesia. 

Postoperative management included continuous rate infusion of lactated Ringer’s solution, buprenorphine 0.005 mg/kg IV four times daily (Bupresol Multidose^®^, CP-Pharma, Ostlandring, Burgdorf) and pantoprazole 1 mg/kg IV twice daily (Pantoprazol^®^, Hexal, Ljubljana, Slovenia). Prednisolone was reduced to 1 mg/kg once daily to reduce the risk of post-operative wound infection and otitis externa was topically treated using silver sulfadiazine suspension (Flammazine^®^, Alliance, Barcelona, Spain) and hypochlorous acid (Granudacyn^®^, Mölnlycke, Dusseldorf, Germany). The dog improved and was discharged three days after surgery with intermittent spontaneous knuckling in the right thoracic limb, improvement of head and cervical pain and persistent bilateral vision loss. Ten days after surgery, prednisolone was increased again to an immunosuppressive dose of 2 mg/kg once daily and a standard cytarabine protocol (ARA-cell^®^, Stada, Bad Vilbel, Deutschland) was started with a dose of 50 mg/m^2^ SC every 12 h for two days. 

The dog remained stable for two weeks after surgery, then started to have generalized epileptic seizures that were treated with phenobarbital 3 mg/kg PO twice daily (Phenoleptil^®^, Dechra, Oudewater, The Netherlands), and finally developed cognitive decline and non-ambulatory tetraparesis and was therefore euthanized four weeks after surgery. 

**Figure 4:**
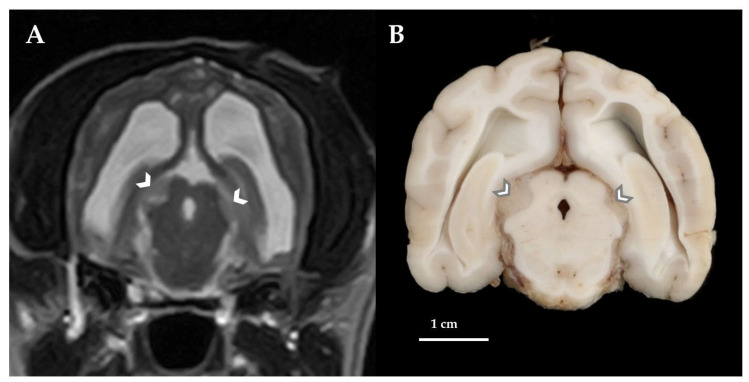
Post-mortem MRI, transverse T2W (**A**) and frontal brain section, formalin-fixed (**B**) of the dog’s brain at the level of the midbrain. Post-mortem MRI demonstrated a resolution of the SFA, a moderate hydrocephalus internus and an enlargement of the midbrain lesions (arrowheads, **A**). On necropsy, the midbrain lesions revealed to be macroscopic tumor foci arising from the subarachnoid space (arrowheads, **B**). The left side of the patient is on the right side of the image.

Post-mortem MRI was performed obtaining the same sequences as described for the first MRI excluding post-contrast images and showed a complete resolution of the SFA, a hydrocephalus internus with dilation of olfactory recesses, periventricular FLAIR and T2W hyperintensities consistent with periventricular oedema and a flattening of the interthalamic adhesion as well as an enlargement of the midbrain (Figure 4A) and thalamic lesions that had already been recognized on the first MRI. 

**Figure 5:**
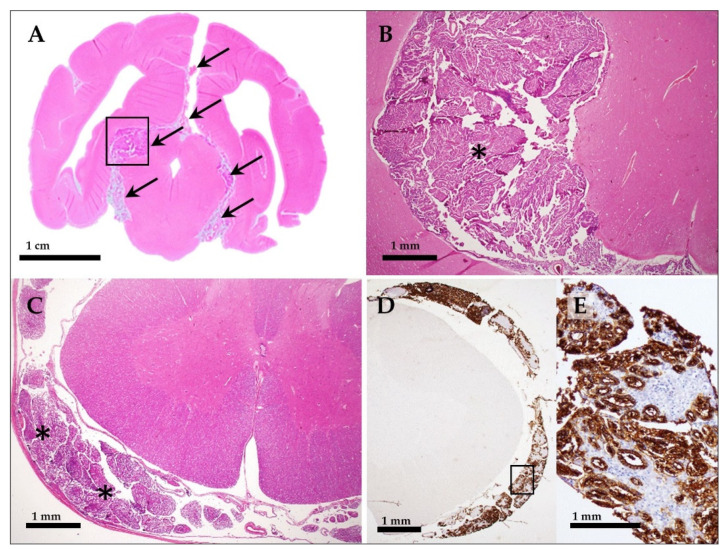
Diffuse, invasive choroid plexus carcinoma involving the entire central nervous system of the dog. Histopathological sections of the formalin fixed brain (**A**,**B**) and spinal cord with meninges and spinal nerve roots (**C**–**E**) on HE and immunohistochemistry for detection of Kir7.1 (**D**,**E**). In (**A**), a transverse brain section at the level of the rostral colliculi is shown, highlighting the subgross tumor foci (arrow, compare to Figure 4A,B). The detail enlargement of the tumor (asterisk), corresponding to the location indicated by the black rectangle in (**A**), is shown in (**B**). Spinal cord with tumor invasion (asterisks) of spinal nerve roots (**C**–**E**). Images in (**D**,**E**) show immunohistochemical detection of Kir7.1 in epithelial tumor cells (brown reaction product) within the spinal cord meninges and surrounding spinal nerve roots (**D**). The image in (**E**) shows a detail enlargement of the location indicated by the black rectangle in (**D**).

Subsequently, a full post-mortem examination with pathologic-anatomical inspection of a broad spectrum of different organs and tissues was performed (including brain, spinal cord, skin, peripheral lymph nodes, bone marrow, peripheral nerves, musculoskeletal system, joints, skull with nasal cavities and sinuses, eyes, external ear canals and middle ears, oral cavity, pharynx, larynx, neck organs, lung, heart, esophagus, stomach, intestine, liver, pancreas, spleen, kidneys, urinary bladder, vessels, and endocrine glands). Necropsy confirmed the hydrocephalus internus and revealed multiple, focally extended dural hemorrhages at the level of the left parietotemporal (corresponding to the previous SFA and craniotomy-site) and olfactory lobes as well as osteolytic lesions of the sphenoid bone. On transverse sections of the unfixed brain multiple, moderately demarcated, tan-opaque tissue masses of soft consistency arising from the subarachnoid space at the level of the midbrain (Figure 4B), thalamus and cerebellum were recognized. The remaining organs and tissues did not display relevant gross-alterations, except for agonal changes in the lung including edema and emphysema. For histopathology, tissue samples were routinely fixed in 10% neutrally buffered formalin and processed for standard paraffin-embedding and hematoxylin–eosin (HE) staining. Histopathology samples of the brain and spinal cord included frontal sections at the level of the olfactory cerebrum, the basal nuclei, the thalamus and lateral ventricles, the cerebellum, the brainstem and the fourth ventricle, as well as cervical, thoracic, lumbar and sacral locations of the spinal cord. Histopathological examination of the central nervous system revealed a diffuse, expansively and infiltratively growing neoplasm forming strands and nests of moderately atypical plexus-like epithelium supported by a fibrovascular stroma involving the surface of the lateral and fourth ventricles and leptomeninges of the entire brain and spinal cord (Figure 5A–C). The cuboidal to columnar neoplastic cells were arranged in single or multiple layers and had a weakly basophilic, granular cytoplasm and ovoid, large, central nuclei with densely granular chromatin and rarely recognizable nucleoli. The mitotic rate was two mitotic figures in 10 high-power-fields (HPF) of view (HPF area = 311.72 × 10^3^ µm^2^). There was neoplastic invasion of the sphenoid bone and the adjacent brain parenchyma at multiple sites including the cerebral cortex, cerebellum and brainstem. Neoplastic cells displayed a strong immunoreactivity against the CPT marker Kir7.1 [16] (Figure 5D) and a weak heterogenous immunoreactivity for pan-cytokeratin (data not shown). Immunohistochemical (IHC) detection of Kir7.1 or for detection of pan-cytokeratin, respectively, was performed, exactly as described previously in detail [17,18] and summarized in Table 1. Based on these findings, a diffuse, infiltrative choroid plexus carcinoma with disseminated meningeal infiltration, drop metastasis and a secondary hydrocephalus internus was diagnosed. Gross- and histopathological examination of other tissues and organs, including peripheral lymph nodes, eyes, intraorbital parts of the optic nerves, middle ears and external ear canals revealed no evidence of lympho- or hematogenic tumor metastasis or perineural spread/infiltration outside of the central nervous system. Additional (unrelated) histopathological findings comprised marked myocardial and pancreatic lipomatosis and mild, non-purulent exudation and subtle lymphohistiocytic infiltration of the middle ear mucosa and the external ear canal.

## 3. Discussion

Unusual CPTs that lack the typical MRI features of intraventricular neoplastic mass lesions rarely have been reported in dogs. In two of those cases, multifocal cyst-like lesions and meningeal changes consistent with disseminated meningeal metastasis caused by a CPC have been described as unusual MRI features [13,14]. MRI in our dog demonstrated multifocal changes consisting of cyst-like lesions and meningeal involvement, resembling the findings that have been previously described. 

The most unusual finding in our case was a compressive SFA, a MRI feature that is commonly associated with subdural empyema [20], traumatic [21] or non-traumatic hematoma [22,23] or as the result of overshunting after ventriculoperitoneal shunt insertion in dogs [24] but has never been described as MRI feature in CPCs. At the time of presentation, the underlying cause of this lesion was unclear. A subdural empyema or hematoma were suspected to be less likely as imaging characteristics in our case differed from what has been previously described in these etiologies [20,25]. Though subdural empyema might demonstrate the same signal intensities on T2W, T1W and FLAIR images as the SFA in our dog, in empyema, a heterogenous or peripheral contrast enhancement of the subdural content and an additional leptomeningeal enhancement is present in 100% and more than 50% of dogs, respectively [20], both of which was missing in our dog. The SFA in our dog did not demonstrate a signal void in T2* images, a feature that is normally suspected in chronic hematoma due to the presence of hemosiderin [25,26] though—at least in humans—chronic subdural hematoma might lack this MRI feature [26]. 

Cystic neoplasia, SFA secondary to meningitis, which has been commonly described as a complication of bacterial meningitis in infants [27], parasitic cysts, or congenital malformation were considered possible differential diagnoses for the SFA in our dog. In contrast to empyema, post-meningitis SFAs are mostly composed of clear CSF-like fluid and are thought to arise from CSF blockade at the level of the arachnoid villi secondary to meningitis [27]. 

CSF analysis in this dog provided no additional information to favor either an inflammatory or neoplastic disease, as the marked mononuclear pleocytosis and increased protein could have been also related to meningoencephalitis. The total cell count in CPTs has been described as variable, ranging from 1 to 260 cells/µL [3]. In terms of differentiating CPP from CPC a marked increased CSF protein of more than 80 mg/dL has been demonstrated to be significantly associated with CPC, which was compatible with the result in our dog. At time of CSF sampling, no atypical cells could be identified on CSF cytology that are present in around 50% of CPCs in dogs [3] and therefore might provide a diagnostic value for ante-mortem diagnosis [28]. 

MRI of the whole spine was not performed in our dog. At first presentation, the dog only demonstrated mild cervical and head pain that was explainable by the mass effect caused by the SFA or by the meningeal involvement that is known to be painful, e.g., in terms of meningitis [29]. It therefore remains unclear if spinal meningeal and infiltrative lesions could have already been noted on MRI.

The large extent of the disease in this dog was not identified until histopathology was performed, which revealed a diffuse, invasive CPC that involved the entire CNS including the ventricular system, the meninges of the brain and the spinal cord with infiltration of the adjacent neuroparenchyma. The extensive meningeal involvement in our dog might have been crucial for the development of the SFA in this patient. Meningeal changes caused by metastasis can impair the mechanism of CSF absorption in the same way as it is described in post-meningitis SFAs in humans, and therefore, the same pathomechanism might have caused the SFA in our dog. Impaired CSF production and absorption have also been suspected as an underlying cause in the development of cyst-like lesions in CPTs [5] that have been rarely reported in dogs [13,30,31], in up to 34% of humans [5] and that were also present in our dog.

Post-mortem examination was also able to identify the real origin of the midbrain and thalamic lesions noted on MRI that were believed to be intra-axial in origin due to the absence of characteristic extra-axial signs but appeared to be multifocal macroscopic mass-like tumor foci arising from the subarachnoid space. It has to be mentioned that diagnostic imaging in this dog was performed with a low-field MRI and interpretation of these lesions could have been more straightforward when using high-field MRI. 

Different immunohistochemical markers have been described for CPT in dogs, including cytokeratins, glial fibrillary acidic protein, vimentin, E- and N-cadherin, and β-catenin, but immunoreactivity can be inconsistent [2,16]. Recently, Kir7.1, an inward-rectifier potassium channel, has been described as an excellent marker for CPT in humans and dogs [16] and a strong immunoreactivity of neoplastic cells was also present in the present case. 

Treatment guidelines for primary brain tumors in dogs including CPTs are lacking. Therapeutic options applied in veterinary medicine are often non-specific for tumor types and include palliative treatment, consisting of corticosteroids, analgetic and antiepileptic drugs, chemotherapy, irradiation and surgery [32]. Surgery is the treatment of choice in CPTs in humans and gross total resection (GTR) of the tumor is significantly associated with the time of survival and one of the most important prognostic factors besides histological tumor grade [11,33]. While in CPPs in humans GTR is often curative the prognosis in CPCs is still poor with a 5-year overall survival in 20–58% of people depending on the extent of surgical resection [10]. In veterinary medicine, the majority of studies concerning surgical treatment of brain tumors exist for meningiomas, which are normally much easier to access than intra-axial or intraventricular mass lesions [32]. There are only two reports about surgical treated intracranial CPTs in dogs which were located in the lateral and fourth ventricle and in which a tumor resection was performed via interhemispheric-transcallosal and a telovelar approach, respectively [34,35]. In both cases, the tumor was confirmed as CPP and both dogs demonstrated a favorable outcome with surgery. 

Adjuvant treatment with radiation- or chemotherapy has been shown to improve the overall survival rate in human patients, especially if GTR cannot be achieved, e.g., due to brain infiltration or the presence of disseminated leptomeningeal metastasis [8,10,36]. A diffuse involvement of the meninges of the entire CNS was one of the most striking findings in our dog and resembles disseminated meningeal metastasis in humans that occur in 45% of CPC in infants and is associated with a poor prognosis and shorter survival time [8,11]. Irradiation of the entire neuroaxis is recommended as adjuvant treatment of CPC in all humans older than three years [36,37], while in younger infants chemotherapy is favored due to a high risk of late neurological sequelae in this group of patients [10]. Large studies for irradiation of CPT in dogs are missing and available data for median survival times include other tumor types such as meningioma or glioma that are usually overrepresented in these studies compared to less common CPT [32,38,39,40].

The medical treatment in our dog consisted of corticosteroids, analgetic and antiepileptic drugs after seizure onset, with an adjuvant cytosine arabinoside therapy, which is routinely used for adjuvant treatment in meningoencephalitis in dogs [41] but also as a component in chemotherapeutic protocols for lymphoma [42,43]. Surgical evacuation of the compressive SFA was suggested after MRI based on the current recommendations in veterinary and human medicine for the treatment of other types of subdural lesions such as empyema [20,44] and compressive post-meningitis SFAs in humans [27]. Based on the multifocal nature of the disease and the failure to respond to medical treatment, the prognosis even with surgery was considered guarded to grave. Nevertheless, the owner elected surgical evacuation of the SFA over euthanasia. Post-mortem MRI did not demonstrate a recurrence of the SFA that could have explained the neurological deterioration that the dog developed some weeks after surgery. Clinical signs in CPTs are thought to be most commonly related to a secondary progressive hydrocephalus [45], a finding that is present in around 75% of dogs with CPT [3] and that was also identified on post-mortem MRI in our dog and therefore could have caused the worsening of neurological signs. As an intraventricular mass lesion was missing in our dog that could have caused obstruction of the ventricular system, it seems more likely that the hydrocephalus was caused by impairment of CSF production and absorption secondary to the CPC [5,6]. An effective palliative therapy option for obstructive hydrocephalus internus due to intraventricular neoplasia has been described in four dogs using ventriculoperitoneal shunting [46]. Temporary extraventricular drainage due to hypertensive hydrocephalus and permanent ventricular-peritoneal shunt as adjuvant treatment in CPTs in humans are required in 35% and 17% of children, respectively [36]. Another reason for the neurological deterioration in our patient was most likely the invasive and extensive nature of the CPC and it therefore appears questionable if additional treatments such as ventriculo-peritoneal shunting or irradiation could have been beneficial in this dog. 

## 4. Conclusions

In conclusion, this is the first report of a SFA that was associated with a CPC in a dog that demonstrated extensive, diffuse lesions throughout the entire CNS including disseminated meningeal involvement in the absence of an intraventricular mass lesion. The clinician should be aware of rare variants of unusual CPCs that can be associated with a SFA, cyst-like lesions, generalized meningeal changes and lack the presence of an intraventricular mass. 

## Figures and Tables

**Table 1:** Host, dilution, pretreatment, and source of antibodies used for immunohistochemical detection of Kir7.1 and cytokeratin in the present study.

	Pretreatment	Antibody	Dilution	Source
Kir7.1	Boiling (pH 6)	First antibody: Polyclonal rabbit anti human potassium channel Kir7.1	1:2000	kindly provided by S. Hirose [19]
		Secondary antibody/detection: EnVision FLEX + Rabbit (LINKER) (visualization system for automated IHC-staining systems)	ready-to-use	part N° K800921 Agilent Technologies Inc., Santa Clara, CA, USA
Cytokeratin	Proteinase K (* Code S3020)	First antibody: Monoclonal mouse anti-Human cytokeratin	1:50	* clones AE1/AE3, code No. M3515,
		Secondary antibody: Peroxidase conjugated rabbit anti-mouse immunoglobulins	1:100	* code No. P0161

* Dako Denmark A/S, Glostrup, Denmark.

## Data Availability

Not applicable.

## References

[1] Snyder J.M., Shofer F.S., Van Winkle T.J., Massicotte C. (2006). Canine Intracranial Primary Neoplasia: 173 Cases (1986–2003). J. Vet. Intern. Med..

[2] Higgins R.J., Bollen A.W., Dickinson P.J., Sisó-Llonch S. (2016). Tumors of the Nervous System. Tumors in Domestic Animals.

[3] Westworth D.R., Dickinson P.J., Vernau W., Johnson E.G., Bollen A.W., Kass P.H., Sturges B.K., Vernau K.M., LeCouteur R.A., Higgins R.J. (2008). Choroid Plexus Tumors in 56 Dogs (1985–2007). J. Vet. Intern. Med..

[4] Itoh T., Uchida K., Nishi A., Shii H., Nagayoshi T., Sakamoto H. (2016). Choroid Plexus Papilloma in a Dog Surviving for 15 Months after Diagnosis with Symptomatic Therapy. J. Vet. Med. Sci..

[5] Lin H., Leng X., Qin C., Du Y., Wang W., Qiu S. (2019). Choroid Plexus Tumours on MRI: Similarities and Distinctions in Different Grades. Cancer Imaging.

[6] Wisner E.R., Dickinson P.J., Higgins R.J. (2011). Magnetic Resonance Imaging Features of Canine Intracranial Neoplasia. Vet. Radiol. Ultrasound.

[7] Rickert C.H., Paulus W. (2001). Tumors of the Choroid Plexus. Microsc. Res. Tech..

[8] Mallick S., Benson R., Melgandi W., Rath G.K. (2017). Effect of Surgery, Adjuvant Therapy, and Other Prognostic Factors on Choroid Plexus Carcinoma: A Systematic Review and Individual Patient Data Analysis. Int. J. Radiat. Oncol..

[9] Louis D.N., Perry A., Reifenberger G., von Deimling A., Figarella-Branger D., Cavenee W.K., Ohgaki H., Wiestler O.D., Kleihues P., Ellison D.W. (2016). The 2016 World Health Organization Classification of Tumors of the Central Nervous System: A Summary. Acta Neuropathol..

[10] Wrede B., Liu P., Wolff J.E.A. (2007). Chemotherapy Improves the Survival of Patients with Choroid Plexus Carcinoma: A Meta-Analysis of Individual Cases with Choroid Plexus Tumors. J. Neurooncol..

[11] Meyers S.P., Khademian Z.P., Chuang S.H., Pollack I.F., Korones D.N., Zimmerman R.A. (2004). Choroid Plexus Carcinomas in Children: MRI Features and Patient Outcomes. Neuroradiology.

[12] Bentley R.T. (2015). Magnetic Resonance Imaging Diagnosis of Brain Tumors in Dogs. Vet. J..

[13] Oura T.J., Early P.J., Jennings S.H., Lewis M.J., Tobias J.R., Thrall D.E. (2013). Canine Choroid Plexus Tumor with Intracranial Dissemination Presenting as Multiple Cystic Lesions. Case Rep. Vet. Med..

[14] Hughes J.R., Taylor-Brown F.E., Greville-Heygate O., Constantino-Casas F., Williams D.L., Genain M.A. (2021). Multimodality Characteristics of Multifocal Choroid Plexus Carcinoma with Bilateral Calvarial Defects in a Dog. Vet. Radiol. Ultrasound.

[15] Pelegrini L.F., Silva N.F., Campos O.P.S., Nery C.C., Silva F.M., Lemos R.S., Yamauchi K.C.I., Amude A.M. (2019). Medical Therapy Using Omeprazole in 12 Hydrocephalic Dogs: Clinical, Diagnostic, and Therapeutic Findings. Pesqui. Veterinária Bras..

[16] Choi E.J., Sloma E.A., Miller A.D. (2016). Kir7.1 Immunoreactivity in Canine Choroid Plexus Tumors. J. Vet. Diagn. Investig..

[17] Hasselblatt M., Böhm C., Tatenhorst L., Dinh V., Newrzella D., Keyvani K., Jeibmann A., Buerger H., Rickert C.H., Paulus W. (2006). Identification of Novel Diagnostic Markers for Choroid Plexus Tumors. Am. J. Surg. Pathol..

[18] Blutke A., Knebel J., Brühschwein A., Breuer W., Hermanns W. (2012). Hemangiopericytoma in a Cat: A Case Report. Vet. Med. (Praha)..

[19] Nakamura N., Suzuki Y., Sakuta H., Ookata K., Kawahara K., Hirose S. (1999). Inwardly Rectifying K+ Channel Kir7.1 Is Highly Expressed in Thyroid Follicular Cells, Intestinal Epithelial Cells and Choroid Plexus Epithelial Cells: Implication for a Functional Coupling with Na+,K+-ATPase. Biochem. J..

[20] Forward A.K., Plessas I.N., Guilherme S., De Decker S. (2019). Retrospective Evaluation of the Clinical Presentation, Magnetic Resonance Imaging Findings, and Outcome of Dogs Diagnosed with Intracranial Empyema (2008–2015): 9 Cases. J. Vet. Emerg. Crit. Care.

[21] Yanai H., Tapia-Nieto R., Cherubini G.B., Caine A. (2015). Results of Magnetic Resonance Imaging Performed within 48 Hours after Head Trauma in Dogs and Association with Outcome: 18 Cases (2007–2012). J. Am. Vet. Med. Assoc..

[22] Nykamp S., Scrivani P., Delahunta A., Yu-Speight A., Rus R. (2001). Chronic Subdural Hematomas and Hydrocephalus in a Dog. Vet. Radiol. Ultrasound.

[23] Asakawa M.G., MacKillop E., Olby N.J., Robertson I.D., Cullen J.M. (2010). Imaging Diagnosis—Ceroid Lipofuscinosis with a Chronic Subdural Hematoma. Vet. Radiol. Ultrasound.

[24] Kitagawa M., Sakai T., Kanayama K. (2005). Subdural Accumulation of Fluid in a Dog after the Insertion of a Ventriculoperitoneal Shunt. Vet. Rec..

[25] Motta L. (2021). Suspected Chronic Subdural Haemorrhage as a Sequela of Subdural Empyema in an Irish Setter. J. Small Anim. Pract..

[26] Cramer X.J.A., Rassner X.U.A., Hedlund X.G.L. (2016). Limitations of T2 *– Gradient Recalled-Echo and Susceptibility- Weighted Imaging in Characterizing Chronic Subdural Hemorrhage in Infant Survivors of Abusive Head Trauma. Am. J. Neuroradiol..

[27] Vinchon M., Joriot S., Jissendi-Tchofo P., Dhellemmes P. (2006). Postmeningitis Subdural Fluid Collection in Infants: Changing Pattern and Indications for Surgery. J. Neurosurg..

[28] Pastorello A., Constantino-Casas F., Archer J. (2010). Choroid Plexus Carcinoma Cells in the Cerebrospinal Fluid of a Staffordshire Bull Terrier. Vet. Clin. Pathol..

[29] Webb A.A. (2003). Potential Sources of Neck and Back Pain in Clinical Conditions of Dogs and Cats: A Review. Vet. J..

[30] Patnaik A.K., Erlandson R.A., Lieberman P.H., Fenner W.R., Prata R.G. (1980). Choroid Plexus Carcinoma with Meningeal Carcinomatosis in a Dog. Vet. Pathol..

[31] Lipsitz D., Levitski R.E., Chauvet A.E. (1999). Magnetic Resonance Imaging of a Choroid Plexus Carcinoma and Meningeal Carcinomatosis in a Dog. Vet. Radiol. Ultrasound.

[32] Miller A.D., Miller C.R., Rossmeisl J.H. (2019). Canine Primary Intracranial Cancer: A Clinicopathologic and Comparative Review of Glioma, Meningioma, and Choroid Plexus Tumors. Front. Oncol..

[33] Sun M.Z., Ivan M.E., Clark A.J., Oh M.C., Delance A.R., Oh T., Safaee M., Kaur G., Bloch O., Molinaro A. (2014). Gross Total Resection Improves Overall Survival in Children with Choroid Plexus Carcinoma. J. Neurooncol..

[34] Antonakakis M.G., Carletti B.E., Anselmi C., McGrath S., Minguez J.J. (2022). Use of a Telovelar Approach for Complete Resection of a Choroid Plexus Tumor in a Dog. Vet. Surg..

[35] Lehner L., Czeibert K., Benczik J., Jakab C., Nagy G. (2020). Transcallosal Removal of a Choroid Plexus Tumor From the Lateral Ventricle in a Dog. Case Report. Front. Vet. Sci..

[36] Hosmann A., Hinker F., Dorfer C., Slavc I., Haberler C., Dieckmann K., Knosp E., Czech T. (2019). Management of Choroid Plexus Tumors—An Institutional Experience. Acta Neurochir. (Wien).

[37] Mazloom A., Wolff J.E., Paulino A.C. (2010). The Impact of Radiotherapy Fields in the Treatment of Patients With Choroid Plexus Carcinoma. Int. J. Radiat. Oncol..

[38] Schwarz P., Meier V., Soukup A., Drees R., Besserer J., Beckmann K., Roos M., Rohrer Bley C. (2018). Comparative Evaluation of a Novel, Moderately Hypofractionated Radiation Protocol in 56 Dogs with Symptomatic Intracranial Neoplasia. J. Vet. Intern. Med..

[39] Spugnini E.P., Thrall D.E., Price G.S., Sharp N.J., Munana K., Page R.L. (2000). Primary Irradiation of Canine Iintracranial Masses. Vet. Radiol..

[40] Mariani C.L., Schubert T.A., House R.A., Wong M.A., Hopkins A.L., Barnes Heller H.L., Milner R.J., Lester N.V., Lurie D.M., Rajon D.A. (2015). Frameless Stereotactic Radiosurgery for the Treatment of Primary Intracranial Tumours in Dogs. Vet. Comp. Oncol..

[41] Cornelis I., Van Ham L., Gielen I., De Decker S., Bhatti S.F.M. (2019). Clinical Presentation, Diagnostic Findings, Prognostic Factors, Treatment and Outcome in Dogs with Meningoencephalomyelitis of Unknown Origin: A Review. Vet. J..

[42] Scott-Moncrieff J.C.R., Chan T.C.K., Samuels M.L., Cook J.R., Coppoc G.L., DeNicola D.B., Richardson R.C. (1991). Plasma and Cerebrospinal Fluid Pharmacokinetics of Cytosine Arabinoside in Dogs. Cancer Chemother. Pharmacol..

[43] Marconato L., Bonfanti U., Stefanello D., Lorenzo M.R., Romanelli G., Comazzi S., Zini E. (2008). Cytosine Arabinoside in Addition to VCAA-Based Protocols for the Treatment of Canine Lymphoma with Bone Marrow Involvement: Does It Make the Difference?. Vet. Comp. Oncol..

[44] Muzumdar D., Biyani N., Deopujari C. (2018). Subdural Empyema in Children. Child’s Nerv. Syst..

[45] Witten A.J., Mendenhall S.K., DeWitt L.S., Vortmeyer A., Cohen-Gadol A. (2021). Cerebellopontine Angle Primary Choroid Plexus Carcinoma Present in an Adult: Case Report and Literature Review. Cureus.

[46] Orlandi R., Vasilache C.G., Mateo I. (2020). Palliative Ventriculoperitoneal Shunting in Dogs with Obstructive Hydrocephalus Caused by Tumors Affecting the Third Ventricle. J. Vet. Intern. Med..

